# Surface Coating-Modulated Phytotoxic Responses of Silver Nanoparticles in Plants and Freshwater Green Algae

**DOI:** 10.3390/nano12010024

**Published:** 2021-12-22

**Authors:** Renata Biba, Karla Košpić, Bruno Komazec, Dora Markulin, Petra Cvjetko, Dubravko Pavoković, Petra Peharec Štefanić, Mirta Tkalec, Biljana Balen

**Affiliations:** Department of Biology, Faculty of Science, University of Zagreb, Horvatovac 102a, HR-10000 Zagreb, Croatia; renata.biba@biol.pmf.hr (R.B.); karla.kospic@biol.pmf.hr (K.K.); bruno.komazec@biol.pmf.hr (B.K.); dora.markulin@biol.pmf.hr (D.M.); pcvjetko@biol.pmf.hr (P.C.); dubravko@biol.pmf.hr (D.P.); ppeharec@biol.pmf.hr (P.P.Š.); mtkalec@biol.pmf.hr (M.T.)

**Keywords:** silver nanoparticles, plants, green algae, growth, photosynthesis, oxidative stress, gene expression, protein expression

## Abstract

Silver nanoparticles (AgNPs) have been implemented in a wide range of commercial products, resulting in their unregulated release into aquatic as well as terrestrial systems. This raises concerns over their impending environmental effects. Once released into the environment, they are prone to various transformation processes that modify their reactivity. In order to increase AgNP stability, different stabilizing coatings are applied during their synthesis. However, coating agents determine particle size and shape and influence their solubility, reactivity, and overall stability as well as their behavior and transformations in the biological medium. In this review, we attempt to give an overview on how the employment of different stabilizing coatings can modulate AgNP-induced phytotoxicity with respect to growth, physiology, and gene and protein expression in terrestrial and aquatic plants and freshwater algae.

## 1. Introduction

Among a variety of applied nanomaterials, silver nanoparticles (AgNPs) attract a lot of attention due to their prominent antimicrobial effects. Therefore, they have been implemented in a wide range of commercial products such as industrial, household, and healthcare-related items, medical devices, textiles, food packaging, optical sensors, and cosmetics [1,2]. Unceasing production and utilization of AgNPs consequently results in their unregulated release into aquatic as well as terrestrial systems through numerous pathways, which raises concerns over their impending environmental effects [3,4]. 

AgNP stability and susceptibility to transformation upon synthesis are directly related to their surface chemistry, mostly size, charge, chemical functionality, and hydrophilicity [5]. The most important processes that impact bioavailability and biological effects of AgNPs are agglomeration and aggregation, which result in the formation of larger particles, oxidation of elemental silver (Ag^0^) to silver ion (Ag^+^), and subsequent dissolution to dissolved Ag^+^ species, thus modifying the AgNP reactivity [6]. De Leersnyder et al. [7] recently emphasized that stabilization mechanism, aging, and environment significantly influence AgNP stability, as well. Studies have shown that after release into the environment, AgNPs can undergo numerous transformation reactions. These transformations include slow oxidative dissolution by O_2_ and protons, reactions with sulphide, chloride, and organic matter [8,9,10] as well as adsorption of polymers or proteins [6,11]. Observed modifications can have a strong impact on AgNP initial properties and thus reduce their mobility and modify initial concentration (reviewed in Tkalec et al. [1]). In order to prevent AgNP agglomeration and aggregation, different stabilizing coatings, such as carboxylic acids (citrate), polymers (polyvinylpyrrolidone, PVP), polysaccharides (gum arabic, GA), and surfactants (cetyltrimethylammonium bromide, CTAB, and sodium dodecyl sulphate, SDS) are applied during their synthesis. However, coating agents can change AgNP surfaces and thus affect their behavior and transformations in the medium [9]. Moreover, coating agents also determine particle size and shape and influence their solubility, reactivity, and overall stability [12,13].

Plants, being sessile organisms, are prone to accumulation of many environmentally released substances, including AgNPs, and are, in this respect, particularly affected. Therefore, there is an ascending number of studies that investigate potential phytotoxicity of AgNPs. So far, mostly negative impact of AgNP exposure on growth, morphology, and physiology of vascular plants has been reported, although some positive effects have also been found (reviewed in Tkalec et al. [1]). Moreover, it was also discovered that AgNPs often induce oxidative stress and trigger altered gene expression, which, as a consequence, results in changes of protein expression (reviewed in Biba et al. [13]). On the other hand, AgNP toxic effects on the growth and physiology of freshwater algae are far less documented, although they are an important component of water environment and ecosystem. Most of the studies have been performed on the two species of green algae, *Chlamydomonas reinhardtii* and *Chlorella vulgaris*, in which cellular internalization and biotransformation of AgNPs have been investigated [14] as well as AgNP effect on growth and photosynthesis [15,16,17,18,19,20,21,22] (Table 1). AgNP-induced impact on growth and photosynthetic parameters were also examined in *Raphidocelis subcapitata* [23,24,25], *Scenedesmus* sp. [26], and in *Pithophora oedogonia* and *Chara vulgaris* [27] (Table 1). Only a few studies performed on freshwater algae have investigated AgNP influence on oxidative stress induction [21,25] and protein expression [21,28,29].

**Figure 1:**
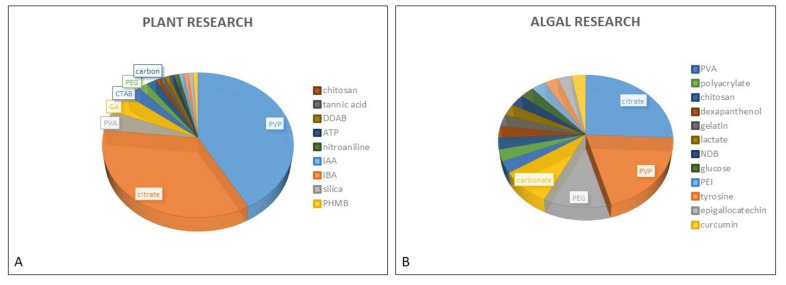
Proportional representation of coatings used for AgNP stabilization in plant (**A**) and algal (**B**) research.

In studies dealing with phytotoxicity of AgNPs, different types of coating agents were used for AgNP stabilization. The PubMed search performed for this review resulted in 16 different coatings used in the assessment of AgNP toxic effects in both plants and freshwater green algae (Figure 1). AgNP stabilization is usually obtained by either steric stabilization, which arises as a consequence of polymer adsorption onto the surface of particles [42], or electrostatic stabilization, which includes surface charge development, usually by physical adsorption of charged species onto the surface [43]. Among nonionic polymer coatings, the most frequently used one is PVP, which has been applied in numerous investigations performed on plants [44,45,46,47,48,49,50,51] and algae [14,20,23]. Besides PVP, polyethylene glycol (PEG) and polyvinyl alcohol (PVA) have also been frequently used for AgNP stabilization in both plant [52,53,54,55,56,57] and algal research [19,20,26,28], while GA, a natural polymer consisting of polysaccharides and glycoproteins, has mostly been utilized in plant studies [10,46,58,59]. Considering the electrostatic stabilization of AgNPs, citrate is the most commonly applied coating that provides a negative charge, and it has been employed in many toxicology studies performed on both plants [47,50,51,60,61,62,63] and algae [20,21,22,29,33,37,40,41,64]. On the other hand, positively charged AgNPs have been scarcely used in plant studies and were usually obtained by application of cationic surfactant CTAB [50,51,65], although didecyldimethylammonium bromide (DDAB) [66] or polyhexamethylene biguanide (PHMB) [67] have also been employed. Cationic polymer polyethyleneimine (PEI) was applied as AgNP coating in the study on freshwater algae *C. vulgaris* [29].

In this paper, we attempt to give an overview on how employment of different stabilizing coatings can modulate AgNP-induced phytotoxicity with respect to growth, physiology, and gene and protein expression in terrestrial and aquatic plants and freshwater algae. Moreover, this is, to our knowledge, the first publication to summarize all aspects of AgNPs toxicity on freshwater algae.

## 2. AgNP Stability in Various Exposure Media

A thorough physicochemical characterization of AgNPs used for toxicological investigations is needed both prior and during the experiment, considering that different exposure conditions may affect their size, shape, and surface electric charge [68,69,70] and, consequently, alter their uptake, toxicokinetics, toxicodynamics, and biological fate [69,71]. Biological media have a high chemical complexity, which is determined by pH, ionic strength, and various concentrations of dissolved organic and inorganic matter. Therefore, it is impossible to correctly predict the form (particulate or ionic) and dose of silver the system is exposed to [72] due to the interactions of AgNPs and the medium that can lead to both agglomeration/aggregation of nanoparticles and their dissolution [73,74,75]. On top of that, chemical or photo-induced reduction of Ag^+^ ions released from the AgNP surface can lead to formation of secondary particles with different characteristics compared to the original ones [70,76,77]. Therefore, understanding AgNP dynamics in exposure medium used for plant and algae treatment plays a key role in interpretation of those toxicological studies.

Colloidal stability of AgNPs in different media used for plant and algal nanotoxicological studies is greatly determined by the composition of the medium itself and the exposure period of the treatment, as discussed in our previous publications [1,13]. Moreover, intrinsic properties of AgNPs (size, shape, and surface charge) also direct their behavior in the environment [11]. Generally, rate of dissolution is higher for smaller uncoated AgNPs in media rich in molecules that tend to complex released Ag^+^ ions [78]. Indeed, plant experiments conducted with uncoated AgNPs revealed significantly higher agglomeration and dissolution rates of AgNPs in tested liquid media used for duckweed (*Spirodela punctata*) [79] and *Arabidopsis thaliana* treatment [80], or sand matrix employed in wheat (*Triticum aestivum*) experiments [81], compared to water treatment of broad bean (*Vicia faba*) [82], lettuce (*Lactuca sativa*), and cucumber (*Cucumis sativus*) [83]. In algal research, significant agglomeration of uncoated AgNPs was also measured in high salt medium (HSM) used for *C. reinhardtii* cultures [17] and BG-11 medium for *C. vulgaris* treatment [32]. Uncoated AgNPs have a negative surface charge due to the presence of hydroxo-, oxo-, or sulfide groups on the surface, which stabilizes them in deionized water. However, existence of counterions in the nutrient media and soil reduces repulsive forces between them and promotes aggregation [9].

Stabilization of AgNPs in a medium can be achieved using surface coatings designed to lower their surface energy, prevent interactions with the environment, and diminish aggregation rates [84,85]. Different routes for AgNP stabilization can be employed, depending on their final application [86]. Citrate, a small monomeric molecule [11,61,87], is commonly implemented as a stabilizer in research of AgNP effects on plants and algae. It provides a highly negative charge at the AgNP surface, ensuring their stabilization through electrostatic means. Stability measurements of citrate-coated AgNPs in water medium using dynamic light scattering (DLS) revealed no significant changes in their size and surface charge in moderately hard water applied for maize (*Zea mays*) and cabbage (*Brassica oleracea*) treatment [88]. On the contrary, changes in AgNP zeta potential connected with the loss of coating and higher dissolution rates in ultrapure water were reported in an experiment with tobacco (*Nicotiana tabacum*) plants [89]. Most of researchers found citrate-coated AgNPs highly unstable in different media with high ionic strength used for plant growth. Significant increase in hydrodynamic diameter, indicating AgNP agglomeration, was observed in liquid half- and full-strength Murashige and Skoog (MS) medium [51,60,90], 1/4 Hoagland medium [91], and in a nutrient solution prepared according to OECD 221 guidelines [62]. Decrease of zeta potential was also reported, indicating the loss of citrate coating [62,92]. Moreover, significant concentrations of Ag^+^ ions were measured both in liquid nutrient media [90,91,93] and in soil [94,95], as a consequence of citrate-coated AgNP dissolution. However, addition of natural polymers, such as Phytagel, stabilized AgNP-citrate in a solid MS medium by encapsulation, which reduced their oxidative changes during exposure of tobacco seedlings [60,61]. On the other hand, AgNP-citrate seem to be quite stable in media used for cultivation of algae. No significant difference was obtained in size and zeta potential values in AgNP-citrate immersed in 10 mmol L^−1^ 3-morpholinopropanesulfonic acid (MOPS) used for *Euglena gracilis* treatment [40], while only minor dissolution was found in BG-11 medium used for *C. vulgaris* [21]. Generally, electrostatically stabilized AgNPs have shown less media-induced modifications when lower ionic strength and higher pH values are applied [7,96]. This could explain their higher stability in algal treatment media compared to media used in plant research. Surfactant molecules, such as positively charged CTAB, are also used in plant AgNP research as electrostatic stabilizers. As with AgNP-citrate, the behavior of AgNP-CTAB changes depending on the medium used for plant treatment and similar trends were observed. AgNP-CTAB was shown to be quite stable in ultrapure water used for treatment of onion (*Allium cepa*) roots [65], but its addition in liquid 1/2 MS medium used for tobacco plants exposure led to rapid agglomeration observed by DLS measurements and transmission electron microscope (TEM) imaging. These findings were additionally corroborated by significant decrease of their zeta potential [51]. Interesting trend was observed with UV-VIS spectrometry in research by Biba et al. [50], where AgNP-CTAB showed good stability in a solid 1/2 strength MS medium used for tobacco germination experiments. However, addition of cysteine, a strong silver ligand, led to rapid dissolution and release of Ag^+^, indicating a fast removal of surface coating and showing that CTAB is a relatively labile ligand.

The use of polymer coatings for AgNP stabilization provides a higher colloidal stability through steric repulsion between the polymer-coated particles [50,87,97,98]. The most frequently used polymer in plant and algal nanotoxicology research is PVP (Figure 1). High stability of PVP-coated AgNPs in plant research has been shown both in ultrapure water [65] and in various nutrient media used for plant growth. The size of PVP-coated AgNPs was found to be constant in 1/2 Hutner’s solution employed for *Landoltia punctata* two-day treatment [99]. Similarly, Jiang et al. [100] reported that 10% Hoagland’s solution used for *Spirodela polyrhiza* treatment had no effect on AgNP-PVP shape and size, although their later research indicated a slight change in AgNP-PVP zeta potential in the aforementioned medium [46]. Yang et al. [101,102] have examined how different environmental factors affect AgNP-PVP stability in media commonly used for growth of rice (*Oriza sativa*). Their results showed that chloride ions, which play an important role in uptake and accumulation of environmental silver (both particulate and ionic), significantly increase AgNP-PVP stability in Hewitt medium by increasing the overall negative charge of NPs, thus enhancing their dispersion [101]. Furthermore, their later research proved AgNP-PVP to be stable in 1/15 Hewitt medium even after the addition of Fe^2+^-EDTA [102]. On the other hand, a couple of studies have demonstrated medium-induced alterations of PVP-stabilized AgNPs. Comparison of DLS results for citrate-, PVP- and CTAB-coated AgNPs in 1/2 strength MS medium used for tobacco treatment showed a slower agglomeration rate for AgNP-PVP compared to AgNP-citrate and AgNP-CTAB, which was also accompanied by the decrease of their zeta potential [51]. This finding indicated increased electrostatic repulsion between nanoparticles. In a similar study, AgNP-PVP were found to be less stable and prone to dissolution in the solid 1/2 MS medium in comparison to AgNP-CTAB [50]. On top of that, addition of cysteine led to their rapid agglomeration coupled with additional dissolution and formation of silver clusters from dissolved Ag^+^ [50]. Stabilization of AgNPs by another type of steric molecule, GA, successfully protected AgNPs against aggregation and dissolution in ultrapure water used for treatment of Italian ryegrass (*Lolium multiflorum*) [10], and in 10% Hoagland medium used for exposure of *S. polyrhiza* [58,100]. Similar stabilizing capabilities were also observed for PEG-coated AgNPs that exhibited no significant changes in size in 1/4 Hoagland medium during *A. thaliana* treatment [52]. Stability of polymer-coated AgNPs was also examined in algal research. PEG-, PVP-, and chitosan-coated AgNPs retained the same size and charge in MOPS used for *C. reinhardtii* treatment even after cysteine addition, confirming their excellent stabilization against dissolution in the medium [20]. On the other hand, in a medium used for *R. subcapitata* cultivation and treatment, AgNP-PVP showed high agglomeration rate that was mitigated with the addition of a commercial humic substance, which provided electrostatic repulsive forces and decreased their zeta potential [23]. When three organic ligands with different numbers of phenol structures were used as AgNP coatings in toxicological studies on algae *R. subcapitata*, differences in their stability were observed in the Elendt M4 medium used for algal growth [25]. The highest rate of aggregation was obtained with tyrosine-AgNPs, followed by epigallocatechin gallate-AgNPs, while curcumin-coated AgNPs showed no signs of aggregation. Observed differences were attributed to different coating materials [25], proving that thorough characterization and stability analyses are imperative for accurate interpretation of nanotoxicological data.

All these findings show that AgNP behavior in the nutrient media is far from predictable. This becomes even more complicated when plants or algae are added to the media. Interaction of AgNPs with the biomolecules present in biological environment (nucleic acids, proteins, lipids, etc.) can lead to the formation of the surface corona [70,83] that can reverse AgNP surface charge [103]. These processes can either stabilize AgNPs or result in their increased aggregation and dissolution rates, depending on the AgNP intrinsic characteristics [73,97]. However, information on the AgNP corona formation in plant and algae is scarce due to the lack of published studies. Considering the emerging interest in application of nanotechnology in the agriculture [104], AgNP modifications due to the surface corona formation should be a focus of any future nanotoxicological studies.

## 3. Silver Uptake and Effects on Growth and Morphology

**Figure 2:**
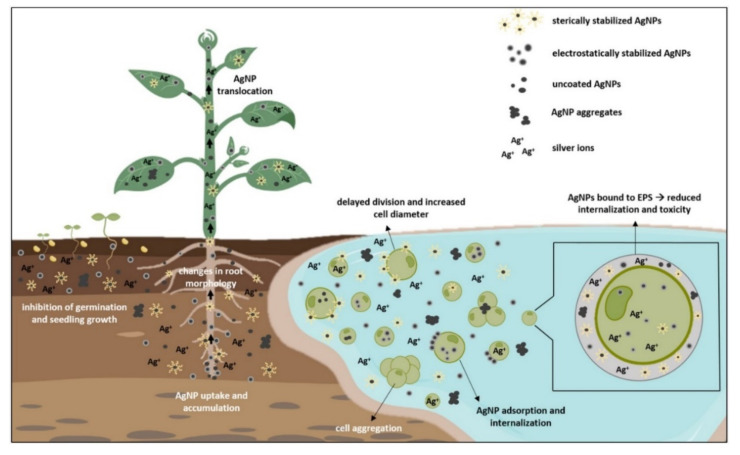
Uptake of differently coated and uncoated AgNPs in plants and freshwater algae and their effects on growth and morphology. EPS—extracellular polymeric substances. Figure was created with BioRender.com. Accessed on 24 November 2021.

Seed germination represents the first and the most crucial step for plant growth and the overall crop yield [105]. It is the most sensitive stage of plant ontogenesis, heavily susceptible to various environmental factors, such as AgNP exposure, that can modulate metabolic processes during germination and ultimately affect plant growth [106]. To assess AgNP effects on seed germination and early growth, most of the conducted studies examined germination percentage and rate, root, and shoot elongation, plant morphology, and changes in biomass [1,50,60,107]. Results showed both positive and negative effects, depending on the plant species, exposure method, and characteristics of AgNPs (reviewed in Tkalec et al. [1]). Another important factor determining AgNP phytotoxic effects is their uptake. The main route of AgNPs entry into the plant cell occurs through the pores in the cell wall [74,108]. Their further translocation occurs by endocytosis and through plasmodesmata [109,110]. AgNP movement and effects are highly dependent on the plant growth stage. If taken up by roots of seedlings or adult plants, AgNPs can penetrate the vascular tissue and reach the stems and leaves (Figure 2), where they can cause further damage [111]. If AgNPs enter the seeds during imbibition period, they can move to embryonic cells and in that way cause long-term effects for the plant [112]. Properties of AgNPs are determined mostly by their size and surface coating that play an important role in AgNP uptake and modulate their effects on germination and development.

Electrostatically stabilized citrate-coated AgNPs showed higher potential for medium-induced modifications, consequently leading to their pronounced phytotoxic effects. In a study by Pokhrel et al. [88], it was reported that AgNP-citrate significantly inhibited seed germination of cabbage and maize, but the effects on root elongation were found to be species-dependent. AgNP-citrate had no effect on maize root growth, but it inhibited growth of cabbage roots. Authors attributed this variance in results to the different size of seeds in question because smaller cabbage seeds with greater surface-to-volume ratio were found to be more prone to interaction with AgNPs [88]. Germination of tobacco seeds was also delayed and slower during the treatments with citrate-coated AgNPs, but effects on the seedling growth were shown to be concentration-dependent [60]. Root growth was enhanced at lower tested concentrations but significantly reduced at higher concentrations. In tomato (*Lycopersicon esculentum*) plants, citrate-coated AgNPs- had no effect on germination, but they significantly decreased root elongation, even in the lowest tested dose [113]. Adverse toxic effects of AgNP-citrate on growth of mung bean (*Phaseolus radiatus*) and great millet (*Sorghum bicolor*) were also reported [95]. AgNPs caused necrosis and browning of the root tissue of both plant species that was consequently attributed to accumulated AgNPs in the cells, as confirmed by TEM and X-ray energy dispersion spectroscopy (EDS). An interesting finding was obtained in the research by Geisler-Lee et al. [114], where no initial effects on germination of *A. thaliana* were detected upon exposure to citrate-coated AgNPs; however, negative effects were confirmed and amplified over the next three generations.

Effects of sterically stabilized AgNP-PVP on germination and plant early growth are also adverse. Scherer et al. [115] observed AgNP-PVP internalization in roots of *A. cepa* that led to reduction of germination index and root elongation. A study conducted on wheat seedlings showed that AgNP-PVP negatively impacted root length and fresh mass upon treatment, even though germination percentage and germination rate were not affected [45]. Furthermore, silver content was higher in roots compared to the leaves of treated seedlings. However, TEM images could not confirm AgNP entry to the cells and observed root tip browning was ascribed to AgNPs adsorbed to the root tissue [45]. Different trends were observed in rocket (*Eruca sativa*) seeds; germination of the seed was also not affected upon treatment with AgNP-PVP, but root growth was significantly stimulated [116]. Another study showed similar silver uptake for castor bean (*Ricinus communis*) seedlings exposed to AgNP-PVP and AgNO_3_. However, AgNP-PVP had no significant impact on castor bean seed germination and growth, while ionic silver significantly decreased those parameters [117]. In contrast, in the research of Wang et al. [118], in which AgNP-PVP was localized in the cell wall and intercellular spaces of *A. thaliana* roots, it was found that AgNPs promoted root growth at low concentrations. However, higher concentrations had the opposite effect, suggesting a dose-dependent response. Another hydrophilic molecule used in AgNP stabilization is PVA, which showed detrimental impact on growth and morphology of *L. punctata* with distinct signs of chlorosis [57]. Similar effects were reported for *O. sativa*, where higher doses induced AgNP-PVA penetration through the cell wall. Moreover, restricted root growth, decrease in dry weight, and significant damage of the cell morphology were revealed [55]. GA is another commonly used steric AgNP stabilizer. Yin et al. [10] found that AgNP-GA inhibited growth of *L. multiflorum* seedlings and significantly changed their root morphology, mainly observed as a lack of root hairs and damaged epidermis and root cap. These results were ascribed to high silver content measured in both roots and shoots of the seedlings. Moreover, detrimental effects of AgNP-GA were not mitigated with the addition of cysteine and were far more pronounced than the effects of ionic silver applied at same concentrations [10].

Only a couple of research groups compared differently coated AgNPs in the same experimental setup in an attempt to deduce the impact of a particular stabilizing agent (reviewed in [13]). Treatment with AgNP-GA generated deleterious results in germination and early growth of eleven wetland plants, which was not observed upon AgNP-PVP exposure [119]. These findings, however, cannot be assigned completely to the coating molecule, since applied AgNPs had different sizes (20 nm for AgNP-PVP and 6 nm for AgNP-GA). Pereira et al. [120] reported that AgNP-PVP and AgNP-citrate caused harmful effects on *Lemna minor* upon treatment, but their mechanisms differed; AgNP-PVP affected growth rate, while AgNP-citrate induced chlorosis. Comparison of effects of uncoated AgNPs, AgNP-PVP, and AgNP-citrate on two developmental stages of bryophyte *Physcomitrella patens* revealed higher growth inhibition of protonema and leafy gametophyte in AgNP-citrate treatment compared to AgNP-PVP [121]. Discrepancy in effects between two developmental stages was found among the treatments with uncoated and citrate-coated AgNPs, i.e., uncoated AgNPs had higher impact on protonemal stage, while AgNP-citrate affected gametophyte stage more, which correlated with a significantly higher Ag uptake in the gametophyte tissue. This was attributed to higher rates of AgNP-citrate dissolution in the used medium over time [121]. In research conducted on tobacco seedlings, uptake of silver was similar for PVP- and CTAB-coated AgNPs and AgNO_3_ [50]. However, germination tests and measurements of root length and fresh and dry weight revealed significantly higher toxicity of AgNP-CTAB compared to AgNP-PVP and even ionic silver. Furthermore, harmful effects of AgNP-CTAB were not reduced with the addition of cysteine, and treatment with CTAB alone exhibited similar results. This finding suggests that phytotoxicity of CTAB-coated AgNPs originates from the coating itself. Similar discovery was reported in a study of AgNP-PVP and AgNP-DDAB effects on pea (*Pisum sativum*), where higher doses of AgNP-DDAB and treatment with DDAB itself significantly reduced seed germination and root length, which was not observed in AgNP-PVP treatment [65]. Presented results show differential response of plants during their early development in regard to coatings used in AgNP treatment. To better understand the mechanism of AgNP phytotoxicity, it is imperative to include more coating-dependent studies in the future.

Algal accumulation of AgNPs is an important process of AgNP transport through the aquatic ecosystem [122]. AgNPs can be adsorbed onto the algae surface and/or internalized in the cell due to the porous structure of the cell wall [6,122]. At normal conditions, only particles smaller than 20 nm can enter the algal cell, but during cell division and stress induction, cell wall permeability increases, allowing entry of even bigger sized particles (Figure 2), causing detrimental effects on their growth and morphology [33,123]. Uncoated AgNPs, which are highly unstable in a liquid medium, triggered significant cell aggregation and reduction of *C. vulgaris* viability [18,34]. The addition of different coatings changes AgNP characteristics and subsequently alter its uptake dynamics and overall effects. Citrate-coated AgNPs had no effect on growth of *C. vulgaris* [21] but significantly inhibited growth of *Microcystis aeruginosa* [21,124], showing differential effect of AgNPs on prokaryotic and eukaryotic algae. Romero et al. [22] reported increase in cell diameter and biomass in *C. vulgaris* upon exposure to AgNP-citrate that was attributed to their delayed division rate. Growth reduction was also observed in AgNP-citrate-treated *E. gracilis*, where further analysis showed that toxicity was not particle-specific but rather the combination of Ag^+^ uptake and AgNP adsorption on the cell surface [40]. Upon exposure to PVP-coated AgNPs, growth of *C. reinhardtii* was not disturbed, even though AgNPs were found in the periplasmic space and cytoplasm. Furthermore, comparative experiments with Ag^+^ exposure excluded the possibility of secondary AgNP formation inside the cell, suggesting AgNP entry into algal cell via cellular internalization [14]. On the contrary, IC50 values showed concentration dependent toxicity of AgNP-PVP in *R. subcapitata* (formerly known as *Pseudokirchneriella subcapitata*), which was significantly mitigated with the addition of humic substances that prevent AgNP dissolution [23]. Dose-dependent growth reduction was also measured in *Scenedesmus* sp. treated with AgNP-PVA [26]. A higher toxicity of AgNP-citrate compared to AgNP-PVP toward growth of *R. subcapitata* was ascribed to their different dissolution rates [26,36,37]. In a comparative study by Kalman et al. [33], AgNP-PVP and AgNP-citrate showed similar uptake rates and growth reduction in *C. vulgaris*, whereas AgNP-PEG treatment resulted in lower toxicity, even though its uptake was significantly faster. This effect could be attributed to the existence of extracellular polymeric substances (EPS), a protective layer on algae surface [41]. EPS can promote AgNP aggregation and complex Ag^+^, limiting overall AgNP bioavailability [125,126]. Moreover, EPS negative charge could be the reason for nonuniform algae response toward treatment with differently coated AgNPs [127]. Zhou et al. [41] have examined the role of EPS in *Chlorella pyrenoidosa* treated with AgNP-PVP and AgNP-citrate. Compared to AgNP-citrate, AgNP-PVP had lower cell internalization rate but higher adsorption constant. Further analyses revealed that AgNP-PVP strongly bind to EPS and have milder effect on plasmolysis and membranolysis than AgNP-citrate, whose highly negative charge limited adsorption onto the cell surface. Removal of EPS led to significant increase of AgNP internalization in both AgNP treatments, showing an important role of EPS in AgNP bioaccumulation. Since EPS evidently play an important role in bio–nano interactions, effects of differently coated AgNPs on EPS should be further investigated.

## 4. Oxidative Stress Induction and Mobilization of Antioxidant Machinery

**Figure 3:**
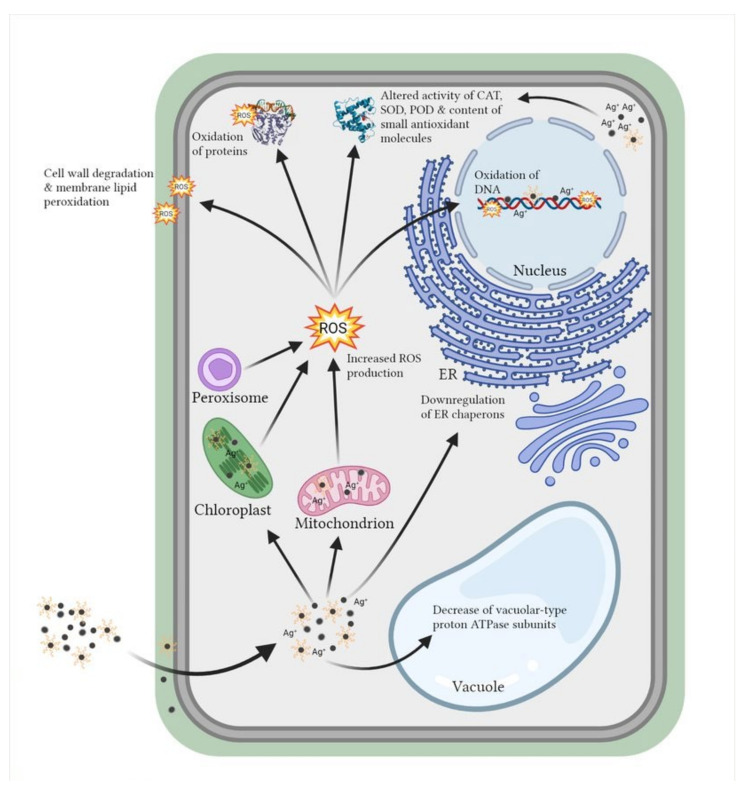
Effect of differently coated AgNPs on plant and algal cells by direct interaction or through ROS formation. ROS—reactive oxygen species, ER—endoplasmic reticulum, CAT—catalase, SOD—superoxide dismutase, POD—peroxidase. Adapted from “Structural Overview of a Plant Cell” by BioRender.com (2021). Retrieved from https://app.biorender.com/biorender-templates. Accessed on 17 December 2021.

Studies have shown that AgNPs are contributing to the production of reactive oxygen species (ROS) [128,129,130]. This can induce oxidative stress in plant and algal cells by combined effects of direct surface oxidation and ability of ROS species to react with important biomolecules (Figure 3), which, under severe oxidative stress, can even lead to the cell death [131]. Indirectly, release of Ag^+^ ions from AgNPs [38,77,132,133] and properties of their coatings [68,133,134] affect AgNP toxicity and additionally contribute to ROS production in promotion of oxidative stress. To elucidate if the extent of oxidative stress is related to AgNP stabilization by coating application, Souza et al. [135] have compared toxicity effects of uncoated and PVP-coated AgNPs on *A. cepa.* Results have demonstrated higher ROS production and more detrimental effects after treatment with uncoated when compared to PVP-coated AgNPs. To the best of our knowledge, such a comparative study on ROS induction in algae has not yet been published. Nonetheless, when *C. vulgaris* was exposed to uncoated AgNPs, a significant increase in ROS formation and lipid peroxidation was detected [32]. The observed effect was ascribed to AgNP-released Ag^+^ ions, which can inhibit different antioxidant enzymes by binding to their essential functional thiol groups [136,137,138]. An independent study of Qian et al. [21] reported that there was no significant change in ROS content in *C. vulgaris* upon exposure to citrate-coated AgNPs. However, significantly increased ROS content and induced oxidative damage was recorded in *R. subcapitata* cells treated with AgNPs coated with L-tyrosine, curcumin, and epigallocatechin gallate [137]. The scarcity of data limits our knowledge on ROS induction upon exposure of algae to AgNPs. Therefore, it is still not clear how differently coated AgNPs influence formation of ROS as mediators of oxidative stress in the AgNP-induced cytotoxicity in algae.

On the other hand, multiple studies have evaluated the effect of electrostatically or sterically stabilized AgNPs on induction of oxidative stress in plants [10,21,55,100,120,139,140]. Nair and Chung [141] demonstrated that exposure to citrate-coated AgNPs led to a dose-dependent increase of H_2_O_2_ content in shoots and roots of rice seedlings, while in situ accumulation of ROS was confirmed in root tips. Similarly, treatment of soybean (*Glycine max*) plants with AgNP-citrate has resulted in increased content of malondialdehyde (MDA), the end product of lipid peroxidation, thus indicating oxidative damage to membrane lipids [139]. Furthermore, elevated ROS production was reported in tobacco seedlings exposed to citrate-coated AgNPs [61], although the application of the same AgNPs to adult tobacco plants failed to induce oxidative stress [47]. This result suggests that plant age and developmental stage also might interfere with AgNP-induced phytotoxicity. In the study by Cvjetko et al. [65], the effects of electrostatically (citrate and CTAB) and sterically (PVP) stabilized AgNPs on roots of *A. cepa* were investigated. The results revealed that PVP-coated AgNPs had the least toxic effects on cell proteins, membranes, and DNA molecule in comparison to citrate- and particularly CTAB-coated ones. Moreover, Biba et al. [50], reported that CTAB-coated AgNPs had higher toxic effect on tobacco seedlings compared to AgNP-PVP. The stronger effect of electrostatically coated AgNPs on the formation of ROS may be related to their relative instability as discussed earlier. When *S. polyrhiza* was exposed to 6 nm AgNP-GA and 20 nm AgNP-PVP, only after the treatment with GA-coated AgNPs an increased MDA content was recorded. This finding was ascribed to AgNP-GA’s smaller size and thus facilitated uptake compared to PVP-coated ones [100]. However, in pollen of kiwifruit (*Actinidia deliciosa*), Speranza et al. [142] have demonstrated that ROS and extracellular H_2_O_2_ production increased after treatment with PVP-coated AgNPs. Authors suggested that pollen as a reproductive plant organ may behave differently toward AgNP-induced stress than vegetative ones [142].

The balance between overproduction and scavenging of different ROS species in algae and plant cells upon exposure to AgNPs is regulated by antioxidant machinery [117]. Galazzi et al. [139] have demonstrated an increase in superoxide dismutase (SOD) and catalase (CAT) activity in soybean plants upon treatment with citrate-coated AgNPs. It was argued that the activity of both enzymes led to decomposition of H_2_O_2_ to H_2_O and O_2_, thus lowering the H_2_O_2_ content in plant cells. The increase in SOD and peroxidase (POD) activities was recorded in *S. polyrhiza* cells upon exposure to GA- and PVP-coated AgNPs, while CAT activity was significantly elevated only after the treatments with AgNP-GA [100]. Similar findings were reported when castor bean seedlings were exposed to PVP-coated AgNPs, which significantly elevated SOD and POD activity at all applied AgNP concentrations, while CAT activity increased only after the treatment with higher ones [117]. Similarly, an increase in POD activity was reported after the treatment of *L. minor* with citrate- and PVP-coated AgNPs, although AgNP-citrate was found to be more toxic. Interestingly, in the same study, CAT activity remained unchanged in all treatments regardless of the coating properties [120]. Cvjetko et al. [47] have revealed that the activity of antioxidant enzymes showed tissue specific responses. Namely, citrate-coated AgNPs induced CAT activity in roots of tobacco plants exposed to even lower AgNP concentrations, while in leaves, CAT and POD activities increased only after treatments with the highest one. The changes in activities of antioxidant enzymes in response to AgNPs were also reported in algae. Significant increase in SOD activity was found in *C. reinhardtii* cells after exposure to uncoated AgNPs, although POD activity was decreased [31]. Lekamge et al. [25] demonstrated that exposure of *R. subcapitata* to AgNPs stabilized with L-tyrosine and epigallocatechin gallate led to increased CAT activation, while curcumin-coated AgNPs increased CAT activity only after the highest applied concentration. In the study of Qian et al. [21], increased CAT as well as POD activity were also recorded after exposure of *C. vulgaris* to citrate-coated AgNPs, although no significant changes in ROS content were found. This result indicates that *C. vulgaris* efficiently alleviated AgNP-induced ROS overproduction by activation of antioxidant machinery. Glutathione-S-transferase (GST), which can metabolize and inactivate different secondary metabolites such as lipid hydroperoxides, was also found to play a role in defense against ROS toxicity mediated by nanoparticles [143]. Increased GST activity was measured upon exposure of common barley (*Hordeum vulgare*) to uncoated AgNPs [144] as well as in *L. minor* [120] and *R. subcapitata* [25] treated with PVP- and curcumin-coated AgNPs, respectively.

Besides enzymatic mechanism, plants and algae counteract oxidative stress by using small nonenzymatic antioxidant molecules such as proline, glutathione (GSH), and carotenoids in the process of ROS detoxification [100,145,146,147]. GSH acts as a scavenger of ROS [148] and, as a substrate in the synthesis of phytochelatins, can be indirectly involved in heavy metal detoxification [100,149]. Concentration-dependent increase of GSH content upon exposure to both GA- and PVP-coated AgNPs was reported in duckweed *S. polyrhiza*, where it efficiently alleviated oxidative stress, probably by chelating Ag^+^ ions released from AgNPs [100]. Elevated carotenoid content was reported in *O. sativa* exposed to uncoated AgNPs [55], as well as in *C. vulgaris* [22] and tobacco [51,60], after exposure to AgNP-citrate. Carotenoids have an important role in preventing free radical reactions, and an increase in their concentration is one of the cell mechanisms to reduce the ROS toxicity [150]. However, it was also found that PVP- and citrate-coated AgNPs can induce a significant decrease of carotenoid content while simultaneously increasing proline amount in wheat [151] and *O. sativa* [141]. Decrease in carotenoid content may be a result of AgNP-induced toxic effects, which, in turn, damage carotenoid biosynthesis pathways [22,152]. On the other hand, an increase in proline content is considered as a systematic response to metal toxicity since proline is an important osmolyte. It acts as a metal chelator and thus may detoxify AgNP-induced ROS overproduction [153,154,155].

It is known that AgNPs can induce DNA damage either by direct AgNP interaction with DNA molecules or through AgNP-induced ROS formation [128,156,157]. Nevertheless, multiple studies have demonstrated that uncoated or differently coated AgNPs have induced oxidative damage while inducing aneugenic or clastogenic effects. Regarding the clastogenic effects, it was reported that uncoated as well as citrate- and PVP-coated AgNPs induced DNA breaks in *V. faba* [82], tobacco [61,156], and *A. cepa* [65,135,158,159], respectively, while uncoated and PVP-coated ones further induced chromosomal aberrations and increased levels of micronucleus [82,159]. Moreover, concerning the aneugenic outcomes, exposure to uncoated AgNPs as well as to those coated with either citrate or PVP has led to a significant decrease in mitotic index in *V. faba* [82] and *A. cepa* [65,158,159]. However, no genotoxic effects were found upon exposure of *A. cepa* roots to AgNPs coated with PVP [160] or chitosan [161], as well as after exposing tobacco plants to AgNP-citrate [47]. Reported lack of genotoxicity could be a result of longer exposure time during which DNA damage could be repaired. Up to this day, to the best of our knowledge, no studies have evaluated DNA damage to algal species after treatment with silver nanoparticles.

Although oxidative stress plays an important role in the toxicity mechanism of AgNPs, it is still not clear whether production of ROS is a directly or indirectly affected through release of Ag^+^ ions, as, in most of the studies evaluated in this review, detailed analysis of AgNPs stability is missing. Differentially coated AgNPs show different extents of ROS production as well as altered activity of antioxidative enzymes. The overall results suggest that coating-stabilized AgNPs influence plant and green algae response to stressful conditions in time- and concentration-dependent manner, although plant developmental stage can also interfere with the extent of AgNP-induced oxidative stress.

## 5. Impact on Photosynthesis

**Figure 4:**
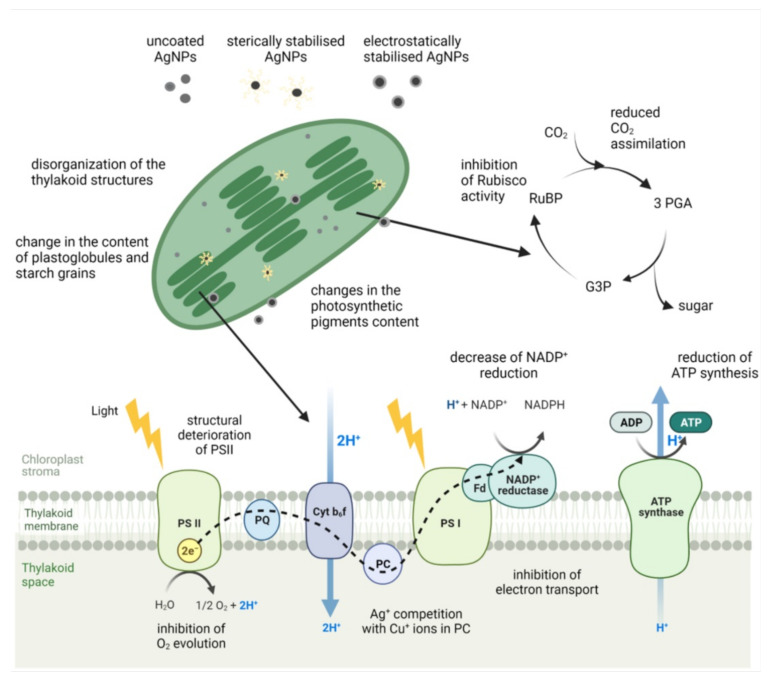
Structural and functional changes of the photosynthetic apparatus in plants and freshwater algae upon exposure to AgNPs with different surface coatings. RuBP—ribulose 1,5-bisphosphate, 3-PGA—3-phosphoglyceric acid, G3P—glyceraldehyde 3-phosphate, PS—photosystem, PQ—plastoquinone, Cyt b6f—cytochrome b6f, PC—plastocyanin, Fd—ferredoxins. Figure was adapted from “Light Dependent Reactions of Photosynthesis” by BioRender.com (2021). Retrieved from https://app.biorender.com/biorender-templates. Accessed on 24 November 2021.

Most research to date shows that photosynthesis, the most important biochemical process on Earth for providing energy and oxygen, is particularly sensitive to AgNPs (Figure 4). Several studies have reported a decrease in chlorophyll content upon exposure to uncoated AgNPs in freshwater algae *C. vulgaris* [18,34], *P. oedogonia* and *Chara vulgaris* [27], and *C. reinhardtii* [31], as well as vascular plants, e.g., rice [141,162] and *A. thaliana* [163]. A decline in chlorophyll content was also observed upon exposure to AgNPs stabilized with different surface coatings, e.g., in freshwater algae *Scenedesmus* after exposure to AgNP-PVA [26], *C. vulgaris* treated with AgNP-glucose [35] and AgNP-citrate [22], and in *A. thaliana* exposed to AgNP-citrate [90]. AgNP-induced reduction of carotenoid content has also been reported upon treatment of *A. thaliana* with AgNP-citrate [90,92] and in *O. sativa* exposed to uncoated AgNPs [141]. The observed decrease in content of chlorophyll *a*, a major photosynthetic pigment, was also found to be accompanied with the inhibition of photosynthetic performance after exposure of fresh water green algae to different AgNPs. Uncoated AgNPs decreased chlorophyll content as well as maximum quantum yield for primary photochemistry and electron transport activity in *C. vulgaris* [32]. Moreover, they also induced structural deterioration of photosystem II (PSII) reaction center, the alteration of the oxygen-evolving complex, and the inhibition of electron transport in *C. reinhardtii* [17]. Inhibited photosynthetic yield was also reported in *R. subcapitata* exposed to AgNP-PVP [23] and in *E. gracilis* upon exposure to AgNP-citrate [39,40]. 

In vascular plants, uncoated AgNPs significantly declined the content of photosynthetic pigments and photosynthetic efficiency in *Arabidopsis* [163]. Moreover, exposure to AgNP-GA [58] and AgNP-PVP [46] reduced chlorophyll content and inhibited maximum quantum yield and electron transport rate in duckweed *S. polyrhiza*. In another duckweed species, *W. globose*, the decrease in chlorophyll content and photosynthetic ability was reported after AgNP-ATP treatment and, less pronounced, after exposure to AgNP-citrate [164]. The inhibition of PSII photochemical reactions has been also found in *Arabidopsis* exposed to AgNP-citrate [90, 92], *Lemna gibba* plants treated with uncoated AgNPs [165], and *V. faba* exposed to AgNP-PVP [49]. These results indisputably indicate that AgNPs induce inhibition of PSII activity, which results in the inhibition of electron transport activity and, consequently, increases production of ROS [34,46]. Moreover, a number of studies reported a correlation between AgNP-induced ROS generation and negative effects on photosynthetic parameters in both algae [18,31,34] and plants [46,54,92,141,166].

Although the exact mechanism of AgNP phytotoxicity is still not fully understood, AgNPs in the cell may dissociate to the toxic Ag^+^ ions [1]. They can competitively replace Cu^+^ ions in plastocyanin, an electron carrier in the photosynthetic electron-transfer chain, resulting in the disturbance of the photosynthetic electron transport and ROS generation [167,168]. Furthermore, Ag^+^ can interact with the thiol group of enzymes of chlorophyll biosynthesis, thus interfering with this process [168]. Another possible explanation for impaired photosynthesis could be diminished transpiration rate and stomatal conductance, resulting in lower rate of gas exchange and reduced CO_2_ assimilation, as found in *L. sativa* [169] and *V. faba* [49] after exposure to AgNP-PVP and in rice after treatment with uncoated AgNPs [162]. Similarly, after exposure of tomato plants to AgNP-PEG, a significant decrease in chlorophyll content and severely disrupted CO_2_ assimilation efficiency were found [54]. Slowing CO_2_ assimilation rate could be linked to AgNP-imposed inhibition of Rubisco activity, as this enzyme showed to be very sensitive to Ag^+^ ions [46]. Consequently, the demand for NADPH and ATP is limited, decreasing energy consumption that, when in excess, induces ROS generation and damage to chloroplasts [46]. Microscopic analyses of chloroplasts confirmed that exposure to various AgNPs disturbs their structure. In a study by Cvjetko et al. [47], chloroplasts in leaves of tobacco plants exposed to AgNP-citrate were found to be smaller than in control plants, although the thylakoid system remained well developed. Yet in another study, citrate-, PVP-, and CTAB-coated AgNPs induced disturbance in the chloroplast ultrastructure of tobacco leaves [51]. The observed difference in the effect of AgNP-citrate may be due to the higher stability of electrostatically stabilized AgNP-citrate in distilled water used as an exposure medium in the former study [47] compared to MS medium applied for plant treatment in the later study [51]. Chloroplasts of *A. thaliana* seedlings treated with uncoated AgNPs appeared to be slightly flatter and the grana lamellae, thinner, and thylakoid membranes became more spread out [163]. Furthermore, chloroplasts with accumulated starch grains as well as with fewer intergranal thylakoids were observed in duckweeds after exposure to sterically stabilized AgNP-GA and AgNP-PVP [100] as well as AgNP-PVA [57].

However, stimulatory effects of AgNPs on photosynthesis have also been found. An increase in chlorophyll content was observed in fenugreek *Trigonella foenumgraecum* after AgNP-citrate exposure [170] as well as in vanilla and sugarcane treated with sterically stabilized AgNP-PVP [171,172]. In seedlings of brown mustard (*Brassica juncea*), upon exposure to AgNP-citrate, increased chlorophyll content was recorded, while photosynthetic quantum efficiency improved, indicating that higher number of reaction centers were in an “open state” to carry out light reaction [173]. Interestingly, in tobacco seedlings, upon exposure to AgNP-citrate, enhanced chlorophyll content was measured, although photosynthetic efficiency remained unaltered [60]. Furthermore, an increase in the carotenoids content after the treatment with uncoated AgNPs was observed in rice [55] and in tobacco exposed to AgNP-citrate [51,60]. A foliar application of uncoated AgNPs stimulated chlorophyll synthesis in the leaves of *T. aestivum*, while in lettuce showed no effects [174]. In *Stevia rebaudiana* treated with AgNP-PVP, the increase in the content of photosynthetic pigments was explained as a consequence of observed increase in N, Mg, and Fe concentrations, since these elements are associated with chlorophyll biosynthesis [175]. Moreover, increase in leaf chlorophyll content correlated with enhanced N and P uptake in *Phaseolus vulgaris* [54] and with increased levels of K, Ca, and S in *Lilium* [176], both treated with uncoated AgNPs. Thus, it can be assumed that positive effects of AgNPs on photosynthetic processes observed in some cases can be attributed to enhanced mineral uptake.

Inconsistent plant responses to AgNPs may be due to the difference in intrinsic properties of differently coated AgNPs used in toxicity studies. However, only a few studies compared the effect of AgNPs with different surface coatings on photosynthetic parameters in the same plant/algal species and test conditions. In algae *C. vulgaris*, inhibitory effects of differently coated AgNPs were compared by measuring chlorophyll content, and the results showed that PEG-coated AgNPs had the least effect, whereas AgNP-PVP and AgNP-citrate had similar inhibitory effect [33]. Navarro et al. [20] investigated the effect of nine differently coated AgNPs (chitosan, lactate, PVP, PEG, gelatin, sodium dodecyl benzene sulfonate, citrate, dexpanthenol, and carbonate) on photosynthetic yield of *C. reinhardtii*. All differently coated AgNPs proved to be toxic, decreasing the photosynthetic yield of algae in a dose-dependent manner. Interestingly, the addition of cysteine, a strong Ag^+^ ligand, completely prevented effects on photosynthetic yield, confirming that Ag^+^ ions play a significant role in the AgNP effect on photosynthesis in algae [15,20,177]. In *C. vulgaris* exposed to citrate- and PVP-coated AgNPs, the concentrations of photosynthetic pigments displayed an increment at lower AgNP concentrations but then significantly decreased at higher concentrations [22]. Different responses to different AgNP doses may be the effect of hormesis, i.e., low doses have a stimulatory effect, while high doses have inhibitory impact [176]. In a study of Liang et al. [121], after exposure of *P. patens* to uncoated AgNPs, content of chlorophylls decreased significantly in protonemata, while the exposure to coated AgNPs resulted with either no significant difference (AgNP-citrate) or increase in chlorophyll content (AgNP-PVP). These results lead to conclusion that surface coatings alleviated the damaging effect of uncoated AgNPs, probably influencing their bioavailability in the exposure medium. The effect of AgNPs with different surface coatings (citrate, PVP, and PEG) was also conducted in *L. sativa*, and the results showed that the transpiration rate and stomatal conductance only diminished in plants exposed to AgNP-PVP [169]. Interestingly, an enhanced photochemical efficiency and increase in all photosynthetic pigments content was observed upon treatments with PVP- and PEG-coated AgNPs, while AgNP-citrate did not cause any significant effect [169]. In the study of Peharec Štefanić et al. [51] on tobacco, the effects of three differently coated AgNPs (citrate, PVP, and CTAB) were cross-compared, and the obtained results showed that the extent of photosynthetic damage depended on the coating, with AgNP-citrate having the least effect. On the other hand, AgNP-PVP and AgNP-CTAB induced more significant decline in the content of chlorophylls and xanthophylls as well as had a strong inhibitory effect on the photosynthetic performance. Authors suggested that milder effects of AgNP-citrate could derive from their fast agglomeration in the exposure medium and, consequently, lower bioavailability. On the other hand, more severe impact of other two AgNP types was probably due to the positive surface charge of AgNP-CTAB and higher stability of AgNP-PVP, resulting in their longer availability for uptake [51]. In the same study, an increase in the nonphotochemical quenching (NPQ) and xanthophyll content related to the inhibition of PSII photochemical reactions was observed in plants treated with AgNP-citrate. Increased energy dissipation through NPQ and downregulation of electron flow can protect PSII against overexcitation and damage [177]. Similar findings have also been found in *Arabidopsis* exposed to AgNP-citrate [91,93] as well as in *L. gibba* plants treated with uncoated AgNPs [164] and *V. faba* exposed to AgNP-PVP [50].

In conclusion, exposure of plants and freshwater algae to AgNPs with different surface coatings can cause both structural changes of the photosynthetic apparatus and functional ones that manifest as a decrease in the content of photosynthetic pigments, as well as an inhibition of photochemical reactions and CO_2_ assimilation. The divergence of the AgNP-induced effects on photosynthesis can be partly attributed to differences in physicochemical characteristics of AgNPs and their bioavailability imposed by different surface coatings. However, the effect of other factors such as plant species, developmental phase, type, and time of exposure should be also considered.

## 6. Changes in Gene and Protein Expression

The application of AgNPs modulates morphophysiological, biochemical, and molecular status of plants and freshwater green algae. In spite of the attention that nanomaterial phytotoxicity attracted in recent years, only limited investigations have been conducted on the molecular level effects of AgNPs in plants, while for studies on green algae, even less information in the literature can be found. To examine the molecular bases of AgNP phytotoxicity, gene and protein expression analyses have been performed in model as well as in different crop plants and only a few species of freshwater green algae.

One of the main mechanisms through which AgNPs mediate their effects involves oxidative stress. Most studies revealed altered expression of genes and proteins encoding for plant cell antioxidant machinery. Nair and Chung [141] observed that the expression level of SOD genes (*FSD, MSD1* and *CSD1*) was induced in shoots and roots of rice seedlings upon exposure to different concentrations of citrate-coated AgNPs. Upregulation of SOD genes, which encode for enzymes that are considered the first line of cellular defense against oxidative stress, was also shown in *A. thaliana* leaves [44] and seedlings [178] upon exposure to AgNP-PVP. Similar findings were also reported in the roots of wheat plants treated with sulfidized AgNPs [179]. Moreover, the studies have shown an upregulation of genes for CAT, also involved in ROS scavenging, in several plant species (rice and tomato seedlings, *Arabidopsis* leaves, and wheat roots) treated with differently coated AgNPs (citrate, PVP, and sulfide) [44,141,179,180]. Furthermore, transcriptional upregulation of ascorbate peroxidase (*APX*) genes was observed in rice shoots upon exposure to citrate-coated AgNPs [141]. This finding, in correlation with higher H_2_O_2_ formation, suggests protections of cells from excess accumulation of H_2_O_2_ through the ascorbate–glutathione cycle, where APX reduces the H_2_O_2_ to H_2_O [141]. AgNP-PVP treatment of *A. thaliana* seedlings [178] and roots [118] also caused upregulation of *POD* genes, which encode another group of antioxidant enzymes. Proteomic analyses confirmed the importance of defense against oxidative stress in plants exposed to AgNPs. Induced protein expression of enzymatic antioxidants including SOD, CAT, and POD was reported in rocket [116] and tomato seedlings [180] upon exposure to AgNP-PVP. Interestingly, in the studies of the impact of the same concentration of AgNP-citrate, upregulation of antioxidant enzymes was recorded in tobacco seedlings [61], but in the leaves and roots of adult tobacco plants, the same proteins were downregulated [89]. These findings indicate that, regardless of the applied AgNP coating and concentration, plant age and developmental stage might have a significant role in response to AgNP-imposed stress. In the studies of freshwater green algae, upregulation of gene and protein expression of antioxidant enzymes was recorded in *C. vulgaris* upon exposure to AgNP-citrate [21], while downregulation of SOD expression was reported in *C. reinhardtii* treated with PEG-coated AgNP [28].

Besides antioxidant enzymes, plant cells can protect themselves against oxidative stress by nonenzymatic molecules such as sulfur-containing compounds, GSH, phenolics, and carotenoids [181,182]. Nair and Chung [80] observed upregulation in the expression level of genes involved in the sulfur assimilation pathway (ATP sulfurylase, *ATPS*), phytochelatin synthesis (phytochelatin synthase, *PCS*), and GSH biosynthesis after exposure of *A. thaliana* to citrate-coated AgNPs. Upregulation was also found for *GST* and glutathione reductase (*GR*) genes. Authors concluded that the differential modulation of genes involved in sulfur assimilation and GSH biosynthesis, *GR*, *GST*, and *PCS*, shows the biological role of these pathways in dealing with AgNP-mediated stress response. Moreover, proteomic analyses of rocket roots demonstrated upregulation of proteins involved in metabolism of sulfur-containing amino acids, methionine, and cysteine [45]. Namely, AgNP-PVP exposure caused the accumulation of a vitamin-B12-independent methionine synthase isozyme involved in the biosynthesis of the methionine and cysteine synthase, a key enzyme in cysteine biosynthesis [45]. Cysteine is a direct coupling step between sulfur and its incorporation into GSH, important in plant stress tolerance to ROS. Upregulation in the expression of key genes of flavonoid and anthocyanin biosynthesis pathway was found in *A. thaliana* plants exposed to AgNP-citrate [183]. Flavonoids are the most abundant compound of phenolics, secondary metabolites that act as free-radical terminators, and anthocyanin commonly serves as a nonenzymatic antioxidant to scavenge free radicals and chelate metals under stress conditions.

Treatments with various AgNPs also had an impact on transcriptional status of genes associated with the plant response to pathogens. In the study of Kaveh et al. [178], exposure of *A. thaliana* seedlings to AgNP-PVP led to downregulation of genes involved in systemic acquired resistance (SAR). SAR is a signaling mechanism that is triggered upon infection by certain pathogens or by mechanical damage and results in thickening of the cell wall and other physiological responses that enhance general plant defenses. In *A. thaliana* exposed to citrate-coated AgNPs, Garcia-Sanchez et al. [183] showed downregulation of genes that are key components of the pathogen-detection pathways that activate SAR and salicylic acid signaling. However, salt stress- and wounding-related genes as well as genes involved in the thalianol biosynthetic pathway, a part of the plant defense mechanism, were found to be upregulated in *A. thaliana* after exposure to AgNP-PVP [178]. Following exposure to Ag_2_S-AgNPs, two miraculin-like protein genes were upregulated in cucumber and wheat leaves [184], while in wheat roots, an enhanced regulation of pathogen-inducible ethylene-responsive element-binding protein was recorded [179]. Treatment with AgNP-PVP upregulated expression of chitinases and pathogenesis-related proteins in wheat seedlings [45]. Similar findings were also reported for tobacco seedlings exposed to AgNP-citrate [61]; however, when adult tobacco plants were exposed to the same treatments, a significant downregulation of the same proteins was recorded [89].

Proteomic studies performed on different plant species showed that AgNPs altered abundance of proteins involved in primary metabolism, suggesting that metabolic adaptation of plants plays an important role in mitigating unfavorable changes in the environment [45,61]. Photosynthesis was found to be seriously affected on both proteomic and genomic level upon exposure of plants to either citrate-coated [61,89,92] or PVP-coated AgNPs [44,45]. Similar studies, performed on freshwater green algae, also revealed the strong impact of AgNP-treatments on photosynthetic proteins. Upon exposure of *C. reinhardtii* to PEG-coated AgNP, the majority of the proteins with enhanced expression were those involved in photosynthesis and Calvin cycle [28]. In the study of Liu et al. [185], in which *Nostoc* sp. was exposed to uncoated AgNPs, differentially expressed proteins related to photosynthesis including phycobilisome, photosynthetic membrane, PSII, and PSI reaction center proteins were found to be affected, with PSII proteins being up-, and PSI proteins downregulated. Upon exposure of *C. vulgaris* to AgNP-citrate, an enhanced expression of Rubisco large chain was recorded, while alpha and beta subunits of ATP synthase were found to be downregulated [21]. All these findings on both plants and green algae suggest that photosynthesis is a cell process particularly affected by AgNP-treatments, regardless of the applied stabilizing agent.

Enhanced expression of proteins involved in glycolysis, an important metabolic pathway responsible for conversion of glucose to pyruvate, was recorded after exposure of tobacco seedlings to AgNP-treatment [61]. In rocket, treatment with PVP-coated AgNPs induced the accumulation of the glyoxalase I enzyme involved in detoxification of methylglyoxal, which is a cytotoxic byproduct of glycolysis that accumulates in cells in response to environmental stresses [116]. Furthermore, AgNPs changed the expression of proteins related to protein folding. In particular, Vannini et al. [116] found the downregulation of two chaperons involved in endoplasmic reticulum (ER)-associated degradation and the decrease of two vacuolar-type proton ATPase subunits in rocket seedlings. These findings indicate that ER and vacuole are targets of the AgNP-PVP action. Similar results were reported in wheat roots, where a strong influence of AgNP-PVP on the ER is suggested due to the decreased levels of three ER-resident proteins [45]. Furthermore, treatment of tobacco seedlings with citrate coated AgNPs also showed changed expression of proteins involved in protein folding [61].

In addition to aforementioned effects, AgNPs can also have an effect associated with hormonal signaling. Most of the auxin-receptor related genes were downregulated in *A. thaliana* exposed to AgNP-PVP [186], while Ag_2_S-NPs upregulated genes involved in ethylene signaling pathway in wheat and cucumber [184]. Both hormones are important for plant growth regulation. Moreover, in the study of Kaveh et al. [178], *A. thaliana* exposure to PVP-coated AgNPs was associated with the downregulation of auxin-regulated gene involved in organ size and ethylene signaling pathway.

In the studies performed on green algae, several distinctive effects in gene and protein expression were recorded. Leclerc and Wilkinson [30] have reported an increase in transcript levels of genes encoding known or predicted components of the cell wall and the flagella after treatment of *C. reinhardtii* with polyacrylate-coated AgNPs. This increase in transcript levels can be explained by the attempt of algae cells to recover AgNP-induced damage to the external structures of the cells. Upon exposure of *C. reinhardtii* cells to PVP-coated AgNP, a significant increase was found for cytochrome c6 (*CYC6*) and ferredoxin-5 (*FDX5*) genes, which was considered to be an indicator of the copper deficiency [122]. Furthermore, after exposure of the same algae to the polyacrylate-coated AgNPs, an increased expression level of the copper transport protein 2 (CTR2) was reported [30]. This finding can be explained by Ag^+^ internalization into cell through Cu(I) transporter. Particularly interesting is the study of Zhang et al. [29], who compared effects of two differently coated AgNPs, negatively charged AgNP-citrate and positively charged AgNP-PEI, on protein expression in *C. vulgaris*, and revealed that citrate-coated AgNPs induced downregulation of mitochondrial function-related proteins, resulting in the disruption of metabolic pathways related to energy metabolism, oxidative phosphorylation, and amino acid synthesis. On the other hand, positively charged AgNP-PEI primarily targeted ribosome function-related proteins and interrupted pathways of protein synthesis and DNA genetic information transmission. These findings suggest that coating applied for AgNP stabilization might induce different response in protein expression within the algae cell.

In conclusion, studies have shown that differently coated AgNPs have impact on gene and protein expression in various plant and algae species. Information obtained from these studies increase our understanding of the mechanisms involved in plant and green algae responses to AgNPs, which is relevant for environmental assessments. However, it is difficult to draw unambiguous conclusions since these studies have been investigated in different species, applied different concentrations of AgNPs with different coatings, and employed different exposure times. Therefore, in order to investigate the role of stabilizing coatings in AgNP-induced phytotoxicity on molecular level and to be able to compare different coatings, it would be useful to conduct a research that implemented differently coated AgNPs in the same experimental setup.

## 7. Conclusions

AgNP behavior in plant and algal exposure systems is dependent on surface coatings. On one hand, they stabilize nanoparticles, but on the other hand, are responsible for their physiochemical modifications, such as changes in aggregation and agglomeration, oxidation states, and dissolution rate of Ag^+^ ions. Surface coating-determined AgNP properties play an important role in AgNP uptake and modulate their effects on germination and development in plants. In algae, EPS plays an important role in AgNP bioaccumulation, which is why effects of differently coated AgNPs on EPS should be further investigated. Oxidative stress is proved to be the one of the major mechanisms of the AgNP-induced phytotoxicity in both plants and algae, although application of certain surface coatings seems to alleviate AgNP-induced ROS formation. The process of photosynthesis, in all its complexity, has been particularly affected by AgNPs, although algae, being unicellular organisms, seem to be more susceptible compared to plants. At the molecular level, gene and protein expression analyses confirmed AgNP-generated induction of oxidative stress and photosynthesis as the most sensitive target of AgNP toxic action, regardless of which coating is applied. However, in order to investigate the role of stabilizing coatings in AgNP-induced phytotoxicity on molecular level and to be able to compare different coatings, it would be useful to conduct a more studies which will implement differently coated AgNPs in the same experimental setup.

## Figures and Tables

**Table 1:** Effects of differently coated AgNPs on fresh water green algae. Studies are listed chronologically according to the number of studies performed on particular algal species.

Algae Species	AgNP Coating/ size (nm)	AgNP Concentration	Exposure Medium/Duration	Investigated Parameters	Findings	Reference
*Chlamydomonas reinhardtii*	carbonate/10 to 200; most particles around 25	10 to 100000 nmol L^−1^	10 mmol L^−1^ MOPS/ 1 and 2 h	photosynthesis	inhibitory effects on photosynthesis	[15]
	carbonate/ 29	0.5–10 μmol L^−1^	10 mmol L^−1^ MOPS/ 1 h	bioaccumulation	Ag content increased with increasing exposure time and AgNP conc., reaching steady state conc. between 10^−5^ and 10^−3^ mol L^−1^ *per* cell	[16]
	uncoated/ 50	1, 5, and 10 mmol L^−1^	HSM/ 6 h	photosynthesis	deteriorating effect on the structural and functional integrity of PSII	[17]
	PEG/ 80 ± 13	2 × 10^–5^ mol L^−1^ 2 × 10^–6^ mol L^−1^	Uspensky medium/ 24 h	photosynthesis	delayed fluorescence induction curves	[19]
	polyacrylate/ 5	0–100 μg L^−1^	4× diluted TAP medium/ 60 min	transcriptome	increased expression of transcript for copper transport protein 2	[30]
	PEG/ 20	0.001–2200 µg L^−1^	Woods Hole MBL medium/ 72 h	protein expression	majority of the proteins with differential expression were upregulated, the majority of which were those involved in thiamine biosynthesis, Calvin cycle, and photosynthesis	[28]
	carbonate/40 ± 0.5, chitosan/25 ± 1.7, citrate/17 ± 0.9, dexpanthenol/456 ± 200, gelatin/52 ± 2.8, lactate/ 35 ± 14.8, NDB/45 ± 3.3, PEG/70 ± 8.3, PVP/84 ± 40.0	0–1000 µmol L^−1^	10 mmol L^−1^ MOPS/ 1 and 2 h	photosynthesis	toxicity was related neither to particle size nor to the coatings	[20]
	PVP/ 11.7 ± 1.9	2 mg L^−1^	tris-acetate-phosphate/ 4, 12, 24, 36, and 48 h	AgNP uptake, distribution, and morphology in algal cells	AgNPs enter the periplasmic space after cellular internalization and sequestration by sulfidation of Ag^+^ ions released from AgNPs by thiolates and sulfides	[14]
	uncoated/ 60–120	0, 1, 5, 10, 30, and 50 mg L^−1^	SE medium/ 24, 48, 72, 96, and 120 h	growth, photosynthesis, and oxidative stress	damaged chloroplasts and inhibited photosynthetic pigments synthesis; inhibited growth; increased ROS production and MDA content; activated antioxidant enzymes	[31]
*Chlorella vulgaris*	uncoated/ 50	0.1, 1, and 10 mg L^−1^	BG-11 medium/ 24 h	viability; oxidative stress	strong decrease in chlorophyll content and cell viability; increased ROS formation and lipid peroxidation	[32]
	citrate/10 PVP/10 PEG/10	AgNP-citrate–9–140 nmol L^−1^ AgNP-PEG– 28–935 nmol L^−1^ AgNP-PVP– 28–93 nmol L^−1^	Jaworski’s medium/ 72 h	growth; chlorophyll content; AgNP accumulation	citrate- and PVP-coated AgNPs showed similar uptake rate and toxicity; AgNP-PEG had the highest uptake rate but the lowest toxicity	[33]
	citrate/ 9–10	9.3, 93, 463, and 926 nmol L^−1^	BG-11 medium/ 24, 48, 72, and 96 h	oxidative stress; gene and protein expression	induction of antioxidant enzymes, unabated photosynthesis at growth-inhibitory AgNP concentration	[21]
	uncoated/ 50 and 100	10, 50, 100, and 200 mg L^−1^	f/2 medium/ 24, 48, 72, and 96 h	cell viability, chlorophyll *a* concentration, ROS formation	negative effect on cell viability and chlorophyll *a* content; increased ROS formation	[34]
	glucose/ 20 ± 5	0.1, 1, 10, 100 µg L^−1^ and 1 mg L^−1^	BBM/ 24 h and 1 week	growth, chlorophyll *a* content, AgNP biodistribution, and subcellular localization	exposure time and dose-dependent growth reduction and decrease in chlorophyll *a* content; internalized AgNPs inside large vacuoles	[35]
	citrate/24, PEI/29	AgNP-citrate -; 71.2 ± 13.6 μg L^−1^, AgNP-PEI -; 51.6 ± 9.6 μg L^−1^	BG-11 medium/ 24 h	protein expression	AgNP-coating electrical property-dependent effects: negative AgNP-citrate regulated mitochondrial function-related proteins; positive AgNP-PEI targeted ribosome function-related proteins and interrupted pathways of protein synthesis and DNA genetic information transmission	[29]
	citrate/ 46.8 ± 3.3	90, 180, 360, 720, and 1440 μg L^−1^	BBM/ 24, 48, 72, and 96 h	growth rate, cell diameter and volume; chlorophyll *a* and *b*, content of pheophytin, carotenoids carbohydrate, total lipids and proteins	altered growth kinetics and cell metabolism expressed in photosynthetic pigments and biochemical composition	[22]
*Raphidocelis subcapitata*	PVP/96	EC50 = 9.9 [7.4–13.2] µg L^−1^	BBM/ 96 h	acute toxicity	AgNP-dose dependent toxicity	[36]
	citrate/14 PVP/15 micron/2000–3500	AgNP-citrate – 3.0 ± 0.7 µg L^−1^ AgNP-PVP – 19.5 ± 6.1 µg L^−1^ micron – 966 µg L^−1^	modified USEPA medium/ 72 h	growth rate inhibition	AgNP-citrate was found to be more toxic than AgNP-PVP; micron-sized particles were less toxic than AgNPs; presence of natural organic matter stabilized AgNPs and reduced toxicity in freshwater	[37]
	alkane material/ 3–8	15 and 30 µg L^−1^	MBL medium/ 48 h	kinetics of uptake and elimination of AgNP in comparison to AgNO_3_	AgNP were not able to penetrate the cells, and Ag accumulation happens through the uptake of Ag ions	[38]
	PVP/ 20	0.1 to 1000 μmol L^−1^	1.36 mmol L^−1^ Ca(NO_3_)_2_, 0.73 mmol L^−1^ Mg(NO_3_)_2_, 1.19 mmol L^−1^ NaNO_3_, 0.20 mmol L^−1^ KNO_3_ in sterile Milli-Q water/4.5 h	photosynthetic efficiency	inhibited photosynthetic efficiency; humic substances alleviated AgNP-imposed toxicity in a dose-dependent matter	[23]
	uncoated/ NM300K–16 ± 5 NM302–176 ± 41 M-AgNP–11 ± 3	NM300K– 2.56–25.6 µg L^−1^, NM302– 0.26–25.6 µg L^−1^, M-AgNP– 5–50 µg L^−1^	modified OCED medium without Fe-EDTA/ 72 h	growth	reduced growth in the following order M-AgNP > NM300K > NM302	[24]
	tyrosine/ 0.56 ± 2.27 epigallocatechin/ 9.27 ± 1.29 curcumin/13.68 ± 0.76	0.020, 0.050, 0.080, 0.110, 0.140, 0.170, 0.200, and 0.230 mg L^−1^	MLA medium/ 24, 48, and 72 h	growth, antioxidant enzyme activities	physicochemical characteristics of the AgNP surface coating plays a major role in determining AgNP behavior in growth medium, toxicity, bioaccumulation, and antioxidant enzyme responses of algae	[25]
*Euglena gracilis*	citrate/47	0–40 μmol L^−1^ for photosynthesis 5 μmol L^−1^ for cell morphology 0–10 μmol L^−1^ for uptake	10 mmol L^−1^ MOPS/ 1 and 2 h for photosynthesis 1 h for cell morphology and uptake	photosynthetic yield cell morphology	photosynthetic yield decreased in a concentration-dependent manner; cell morphology was significantly altered: increased uptake with increasing AgNP concentration up to 2.5 μmol L^−1^ AgNPs	[39]
	citrate/ 38–73	0–40 μmol L^−1^	MOPS/ 1 and 2 h	silver uptake, photosynthetic yield	AgNPs adsorb onto the cell surface and can bind extracellular proteins	[40]
*Pithophora oedogonia*	uncoated/ 10 to 15	0.5, 1, 3, and 5 mmol L^−1^	BBM/ 5, 7, and 10 days	chlorophyll content, chromosomal aberrations	cell wall rupture and degradation, reduction in total chlorophyll content, cytological abnormalities	[27]
*Chara vulgaris*	uncoated/ 10 to 15	0.5, 1, 3, and 5 mmol L^−1^	BBM/ 5, 7, and 10 days	chlorophyll content, chromosomal aberrations	reduction in total chlorophyll content, cytological abnormalities with disturbed metaphase	[27]
*Scenedesmus* sp.	PVA/ 6 to 10	5, 20, 50, 100, and 200 μg L^−1^	COMBO medium/ 72 h	growth, chlorophyll *a* concentration, total lipids	change in cell diameter, reduction in chlorophyll *a* content, enhancement of total lipid production	[26]
*Chlorella pyrenoidosa*	citrate/19.3 ± 6.3 PVP/22.0 ± 6.1	10 mg L^−1^	OECD medium/ various exposure times (0–24 h)	growth inhibition, bioaccumulation, interaction between EPS and AgNPs	AgNP-PVP strongly bind to EPS and have lower uptake and toxicity compared to AgNP-citrate; removal of EPS increases Ag uptake for both AgNP-PVP and AgNP-citrate	[41]

## Data Availability

The data used to support the findings of this study are included within the article.

## Abbreviations

AgNP—silver nanoparticle, APX—ascorbate peroxidase, ATPS—ATP sulfurylase, BBM—Bold’s basal medium, BG-11 medium—medium for blue green algae, CAT—catalase, CSD—CuZnSOD, CTAB—cetyltrimethylammonium bromide, CTR2—copper transport protein 2, CYC6—cytochrome c6, Cyt b6f—cytochrome b6f, DDAB—didecyldimethylammonium bromide, DLS—dynamic light scattering, EDTA—ethylenediaminetetraacetic acid, EPS—extracellular polymeric substances, ER—endoplasmic reticulum, Fd—ferredoxin, FDX5—ferredoxin-5, FSD—FeSOD, G3P—glyceraldehyde 3-phosphate, GA—gum Arabic, GR—glutathione reductase, GSH—glutathione, GST—glutathione-S-transferase, HSM—high salt medium, MOPS—3-morpholinopropanesulfonic acid, MS—Murashige and Skoog, MSD—MnSOD, NDB—Na-dodecylbenzenesulfonate, NPQ—nonphotochemical quenching, PC—plastocyanin, PCS—phytochelatin synthase, PEG—polyethylene glycol, PEI—polyethyleneimine, 3-PGA—3-phosphoglyceric acid, POD—peroxidase, PQ—plastoquinone, PS—photosystem, PVA—polyvinyl alcohol, PVP—polyvinylpyrrolidone, ROS—reactive oxygen species, RuBP—ribulose 1,5-bisphosphate, SAR—systemic acquired resistance, SDS—sodium dodecyl sulphate, SE—sludge extract, SOD—superoxide dismutase, TAP—Tris-acetate-phosphate, TEM—transmission electron microscope, USEPA—U. S. Environmental Protection Agency.
